# Linkage Mapping Identifies the Sex Determining Region as a Single Locus in the Pennate Diatom *Seminavis robusta*

**DOI:** 10.1371/journal.pone.0060132

**Published:** 2013-03-20

**Authors:** Ives Vanstechelman, Koen Sabbe, Wim Vyverman, Pieter Vanormelingen, Marnik Vuylsteke

**Affiliations:** 1 Laboratory of Protistology and Aquatic Ecology, Department of Biology, Ghent University, Gent, Belgium; 2 VIB Department of Plant Systems Biology, Gent, Belgium; 3 Department of Plant Biotechnology and Bioinformatics, Ghent University, Gent, Belgium; Nanjing Forestry University, China

## Abstract

The pennate diatom *Seminavis robusta*, characterized by an archetypical diatom life cycle including a heterothallic mating system, is emerging as a model system for studying the molecular regulation of the diatom cell and life cycle. One of its main advantages compared with other diatom model systems is that sexual crosses can be made routinely, offering unprecedented possibilities for forward genetics. To date, nothing is known about the genetic basis of sex determination in diatoms. Here, we report on the construction of mating type-specific linkage maps for *S. robusta*, and use them to identify a single locus sex determination system in this diatom. We identified 13 mating type plus and 15 mating type minus linkage groups obtained from the analysis of 463 AFLP markers segregating in a full-sib family, covering 963.7 and 972.2 cM, respectively. Five linkage group pairs could be identified as putative homologues. The mating type phenotype mapped as a monogenic trait, disclosing the mating type plus as the heterogametic sex. This study provides the first evidence for a genetic sex determining mechanism in a diatom.

## Introduction

Diatoms (Bacillariophyceae) belong to the Stramenopila, which comprise several microalgal groups dominating primary production in aquatic environments [1]. The diatoms are one of the most diverse and productive groups of algae, with an estimated 200,000 species responsible for almost 20% of global primary production [2]. They are also promising from a biotechnological point of view, and hold great potential for the production of high-value bioproducts such as lipids, pigments and biofuels [3]. The available genomic resources for diatoms have grown rapidly over the past few years [4], [5]. In addition, tools for reverse genetics have been developed [6]. However, sexual reproduction, a key feature of most diatom life cycles [7], has never been demonstrated for the most commonly used model diatoms *Thalassiosira pseudonana* and *Phaeodactylum tricornutum* [3]. This prevents the use of forward genetics to link phenotype to genotype, including the use of mutagenesis and QTL mapping [8].

Life cycles, including sexual reproduction, have been studied in detail for only a minute fraction of known diatom species, but these represent most principal diatom lineages. The diatom life cycle comprises two main stages: a prolonged vegetative stage lasting months to years, which is diploid, and a short sexual stage lasting hours to days [3], [7], [9]. During the vegetative stage of the life cycle, a gradual reduction in cell size takes place, caused by physical constraints imposed by their silica cell wall. This cell wall comprises two parts (or thecae), one of which (the epitheca) is slightly larger than, and overlaps, the other (the hypotheca). During mitosis, each daughter cell inherits one maternal valve (which becomes the new epitheca) and de novo synthesizes a smaller hypotheca. Below a species-specific size threshold (the sexual size threshold, SST) cells become capable of sexual reproduction. Restoration of the maximal cell size generally occurs through sexual reproduction. The zygote matures into an auxospore, which expands to on average two to three times the size of the parental cells. After reaching its maximum size, a new so-called initial cell is formed inside the auxospore envelope, initiating a new round of vegetative multiplication. Because of its crucial role in cell size restoration, sexual reproduction is an obligatory stage in the life cycle of most diatoms.

The raphid pennate diatom *S. robusta* has recently been advocated as a model organism to study diatom biology, and in particular life cycle regulation [3]. Like most pennate diatoms [7], [10]–[12], *S. robusta* has a heterothallic mating system with two mating types (MT^+^ and MT^−^) [13], [14]. After cell pairing, each of the two cells forms two morphologically and behaviorally identical gametes. Subsequent zygote formation and auxospore expansion finally result in two initial cells with a cell length of 64–73 µm. Sexual reproduction is easily induced in cultured strains with a cell size below the SST by adding a suitable mating partner which allows sex to be reliably controlled in mating experiments. However, as in some other pennate diatoms (e.g. *T. tabulata* and *F. delicatissima*) [12], [15], intraclonal reproduction (homothally) can sporadically occur (in *S. robusta* in MT^+^). Additional advantages of *S. robusta* are that the cells are reasonably large (SST ∼50 µm apical cell length) and can grow on surfaces allowing easy non-intrusive monitoring of cell and life cycle stages using inverted microscopy.

To date, genetic maps exist for only a small number of species outside the opisthokont and Plantae lineages. Within the stramenopile lineage, linkage maps have been reported for the brown alga *Ectocarpus* siliculosus [16] and several oomycete species [17]–[20], while a preliminary linkage map has been published for the kelp Laminaria japonica [21].

Experimental evidence suggests that mating type determination in heterothallic pennate diatoms is genetic [7], [15], but how mating type determination is achieved is unknown. Here, we report on the construction of a sex-specific linkage map for *S. robusta* based on AFLP markers [22], [23] and its use to identify the sex determining region in this diatom. We present AFLP markers co-segregating with the mating type phenotype, the genetic structure of the mating type locus and the identification of MT^+^ as the heterogametic sex in *S. robusta*.

## Materials and Methods

### Production of a F_1_ mapping population

The experimental strains were selected from a collection of cryopreserved strains of *S. robusta* publicly available in the BCCM/DCG diatom collection (http://bccm.belspo.be/about/dcg.php). A full-sib (FS) family, containing 116 individual F_1_ progeny, was produced from a cross between strains H73A (DCG 0123) and 96A (DCG 0128), having the MT^−^ and MT^+^ mating type, respectively. Their average cell size at the time of crossing was 37.1±0.4 µm and 26.3±0.3 µm respectively. This difference in cell size between the parental strains allowed to distinguish hetero- from homothallic F_1_ auxospores and initial cells after mating. Cultures were inoculated for dark synchronization [24] by growing them for 3 days at 18°C in a 12∶12 h light:dark regime with cool-white fluorescent lamps at approximately 80 μmol photons m^−2^ s^−1^. At the end of day 3, the dark period was extended for another 18 h, after which cells were mixed by transferring a quarter of the suspended cells of both mating types to a new flask which was then replenished with an equal amount of fresh Guillard's F/2 medium. After another 9 h of darkness, light was switched on again, allowing the mixed cultures to progress synchronously through the different sexual stages. Two days later, 200 F_1_ auxospores and initial cells resulting from heterothallic reproduction (derived from two parental gametangia of unequal size) were picked and transferred to 96-well culture plates containing 0.2 ml F/2 medium.

### Phenotyping: determining the mating type of the F_1_ progeny

After four months of weekly re-inoculating the F_1_ cultures in 24-well culture plates (2 ml), cells reached the SST (∼50 µm). The mating type of each F_1_ progeny was then determined by backcrossing to the parental strains as well as to two other strains (85A and 85B) of known mating type. The mating type of 116 F_1_ progeny could be determined unambiguously, yielding a mapping population of 57 MT^+^ and 59 MT^−^ strains. These strains have been deposited and cryopreserved in the BCCM/DCG culture collection.

### DNA extraction of *S. robusta* cultures

For DNA extraction, cultures were grown in 100 ml culture flasks and harvested in exponential phase. Most culture medium was removed and cells were scraped from the surface (cell scrapers; MLS) in 10 ml of remaining culture medium and centrifuged in 15 ml falcon tubes for 15 minutes at 1000 g (4K15(SIGMA)). After removal of the supernatant, the cells were transferred to a 2 ml Eppendorf tube and centrifuged (15 minutes; 1000 g). Eppendorf tubes with cell pellets were frozen at −20°C.

For DNA extraction, 0.5 g Zirconia/Silica Beads 0.1 mm diameter (BioSpec Products), 0.5 ml TE buffer (10 mM TrisHCl (pH 7.6), 1 mM EDTA (pH 8.0)) and 0.5 ml buffered phenol were added to each sample. The samples were bead-beaten (3×; 85 s; 30 Hz). Each time, the samples were cooled on ice. The tubes were centrifuged (Centrifuge 5415R(EPPENDORF); 4°C; 5 m; 10,000 rpm). The water phase containing the DNA was then transferred to a new 2 ml Eppendorf tube and 0.5 ml PCI (phenol:chlorophorm:isoamyl alcohol  = 25∶24:1v/v) was added. The mixture was centrifuged (10,000 rpm; 5 m; 4°C). The water phase was transferred to a new Eppendorf, 0.5 ml PCI was added again and the mixture was centrifuged. The water phase was transferred to a 1.5 ml Eppendorf. 50 µl 3 M NaAc (pH  = ±5), 1 ml 96% ethanol (−20°C) and 2 µl glycogen (−20°C) were added. The samples were incubated over night at −20°C. The next day, the Eppendorfs were centrifuged (13,000 rpm; 30 m; 4°C). The liquid phase was removed and the DNA pellet became visible. 1 ml 70% ethanol was added and the sample was centrifuged (13,000 rpm; 5 m; 4°C). Ethanol was removed and the sample was again centrifuged (13,000 rpm; 5 m; 4°C). The remaining ethanol was removed with a 200 µl pipet and the pellet was dried for at least 20 minutes. Preheated (55°C; 50 µl) TE buffer (pH 8.0) was added to the pellets and the samples were incubated (20 m; 55°C). DNA of the mapping population was stored at −20°C.

### Segregation and linkage analysis

The 116 offspring were analyzed with 54 *Eco*RI+2/*Mse*I+3 AFLP primer combinations (PC's; Table S1) as described in [23]. Detection of the AFLP fragments was made possible by fluorescent labeling of the *Eco*RI+2 primer in the final selective amplification reaction, and AFLP images were generated using LI-COR automated DNA sequencers. AFLP markers were scored on the basis of relative fragment intensities, using the image analysis software AFLPQuantar*Pro* (http://www.keygene-products.com). AFLP markers heterozygous in MT^+^ parent (96A) and homozygous in the MT^−^, and expected to segregate 1∶1 in the F_1_ generation, were termed MT^+^ specific markers. Markers heterozygous in the MT^−^ parent (H73A) and homozygous in the MT^+^ were termed MT^−^ specific markers. Markers heterozygous in both parents, and expected to segregate 1∶2∶1 in the F_1_ generation were termed “biparental markers”. Whenever feasible, biparental markers were scored co-dominantly (i.e., following a 1∶2∶1 segregation pattern). They were scored dominantly (i.e., conforming to a 3∶1 segregation pattern) when heterozygosity could not reliably be discriminated from homozygosity for the present AFLP marker allele. Each AFLP marker was identified by a specific code referring to the corresponding PC and the estimated molecular size of the fragment in nucleotides as estimated by AFLP-Quantar*Pro*. Linkage analysis and segregation distortion tests were performed using the software package JoinMap 4.0 [25]. The appropriate mapping population type was set to option CP, a population resulting from a cross between two heterogeneously heterozygous and homozygous diploid parents, linkage phases originally unknown. Because for population type CP, the segregation type (SEG) might vary across the loci, a code indicating the segregation type has to be given. The SEG was set to <nn×np> for the MT^+^ markers, <lm×ll> for the MT^−^ markers and <hk×hk> for the biparental markers. The two characters left and right of the ‘‘×’’ in these codes correspond to the AFLP marker alleles of the first and second parent, respectively. Each different AFLP marker allele is represented by a different character. We first ran through a fairly wide range of logarithm of the odds (LOD) thresholds, from 2.0 to 14.0, to obtain a proper view of what might be the best grouping. In general, we decided to use the grouping obtained with a LOD score of 6.0. In a few cases, the grouping obtained at a LOD score of 8.0 and 14.0 was used. Only linkage groups containing at least three markers were considered for map construction. Maps were constructed in three rounds, each producing a linkage map. In this map-building procedure, each map was calculated by using the pairwise data of loci present on the map, with default settings (recombination frequency (REC) <0.4; LOD threshold >1). Once the well-fitting markers (causing a change in goodness of fit smaller than the threshold  = 5) were positioned on the map (after two rounds), the remaining markers were forced onto the map by setting the jump threshold to zero. When the markers in the third map caused a jump in goodness of fit larger than an arbitrary threshold of 10, the second map was selected as the final map, otherwise the third map. Single markers with a segregation ratio in discordance with the flanking markers (i.e., markers showing strong segregation distortion flanked by a number of non-distorted markers) were discarded, and the map construction was repeated. A marker order was not forced on any linkage group during map construction. Recombination frequencies were converted to Kosambi centiMorgans (cM) prior to the map estimation. Linkage groups were constructed using the MT^+^ and MT^−^ markers. Biparental markers were included in both the MT^+^ and MT^−^ linkage maps. This way, homologous linkage groups were identified on the presence of identical biparental markers. Editing the linkage groups was done with the software MapChart [26].

### Mapping the MT locus

The mapping of the mating type phenotype was done in two ways: 1) by including the phenotype as a single marker segregating as a MT^+^ or MT^−^ specific marker, indicated as SEX1 and SEX2, respectively, and 2) by QTL analysis using mixed models as implemented in the QTL menu in GenStat 14 [27]. Because too few homologous linkage groups are identified, QTL analysis was done for the two mating type specific linkage maps separately. In a preliminary search for QTL, we tested the association of individual marker loci with mating type every 5 cM along the genome, using the commonly known simple interval mapping (SIM) procedure. In a second step, we tested for QTL at particular positions after correcting for QTL elsewhere in the genome, as were identified in the preliminary analysis. This procedure is commonly known as composite interval mapping (CIM). The genome-wide type I error rate was set to α = 0.05. The *P* values calculated assume normally distributed errors, when a binomial distribution is more appropriate in the case of mating type. A previous study [28] has shown that applying the mixed model to binary traits is robust and do not result in an excess of low *P* values (i.e. false discoveries) as long as the minor allele frequency of the response variable and the markers is not too low.

## Results

### Segregation analysis and linkage mapping

**Figure 1:**
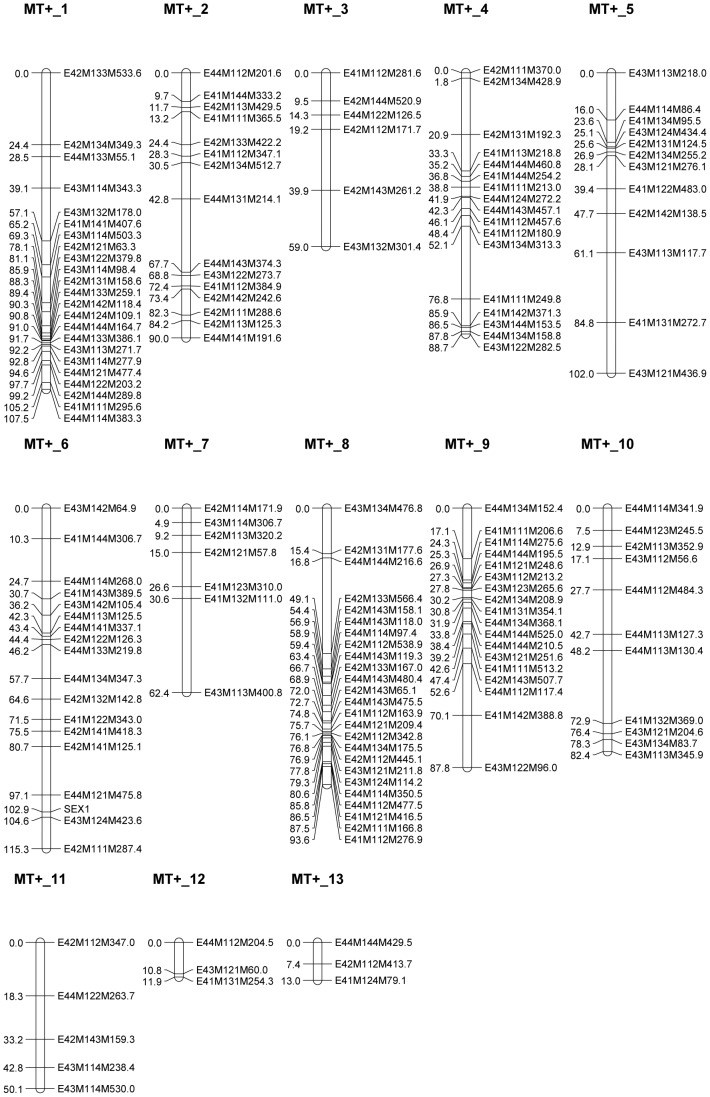
MT^+^ linkage groups of *S. robusta* containing markers originating from parental strain 96A.

**Figure 2:**
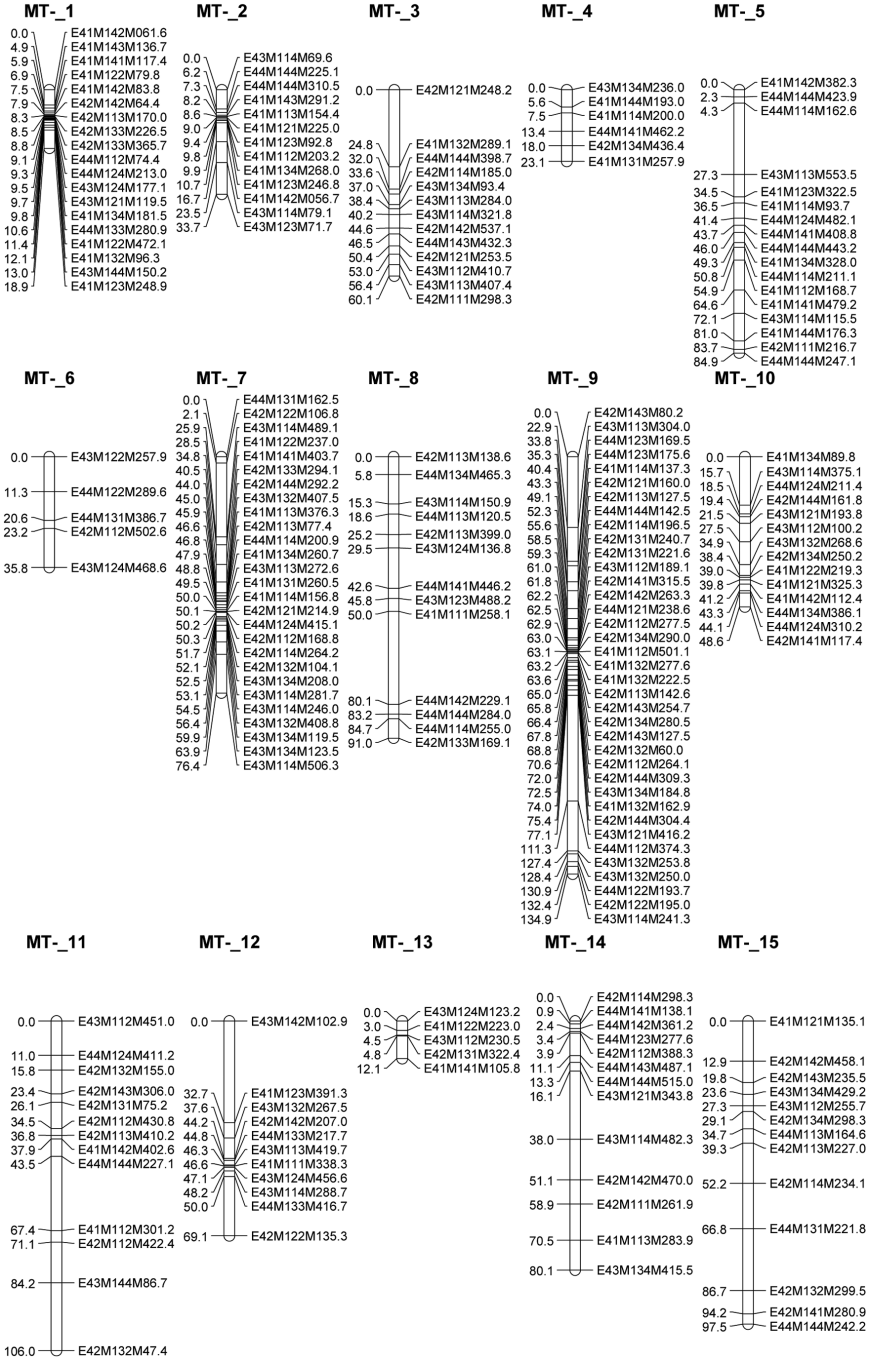
MT^−^ linkage groups of *S. robusta* containing markers originating from parental strain H73A.

A total of 54 *EcoRI*+2/*MseI*+3 AFLP PCs, generating on average 8.6 markers, resulted in a total of 463 AFLP fragments segregating in the 116 F_1_ progeny. In total, 162 MT^+^ and 221 MT^−^ markers were used for the construction of 13 MT^+^ and 15 MT^−^ -specific linkage groups, covering 963.7 cM and 972.2 cM respectively (Figure 1 and 2). Of those marker loci, 28% displayed significantly distorted segregation ratios at the α = 0.05 significance level. As segregation distortion is a normal phenomenon in wide crosses, these markers were not a priori excluded, but evaluated after map construction. Although some larger genomic regions did not reveal any markers (e.g. 32.3 cM in the MT^+^_8 linkage group and 34.2 cM in linkage group MT^−^_9), the median inter-marker distances were relatively low (4.1 and 2.3 cM for the MT^+^ and the MT^−^ linkage maps respectively) (Table 1).

**Table 1:** Statistics for the integrated MT^+^ and MT^−^ linkage maps of *S. robusta*.

	MT^+^	MT^−^
No. of linkage groups	13	15
No. of markers per linkage group		
Min	3	5
Max	25	37
Median	12	13
Mean	12.5	14.7
Total	163	221
Size of linkage groups (cM)		
Min	11.9	12.1
Max	115.3	134.9
Median	87.8	69.1
Mean	74.1	64.8
Total	963.7	972.2
Intermarker distance (cM)		
Min	0.1	0
Max	32.3	34.2
Median	4.1	2.3
Mean	6.4	4.7

**Figure 3:**
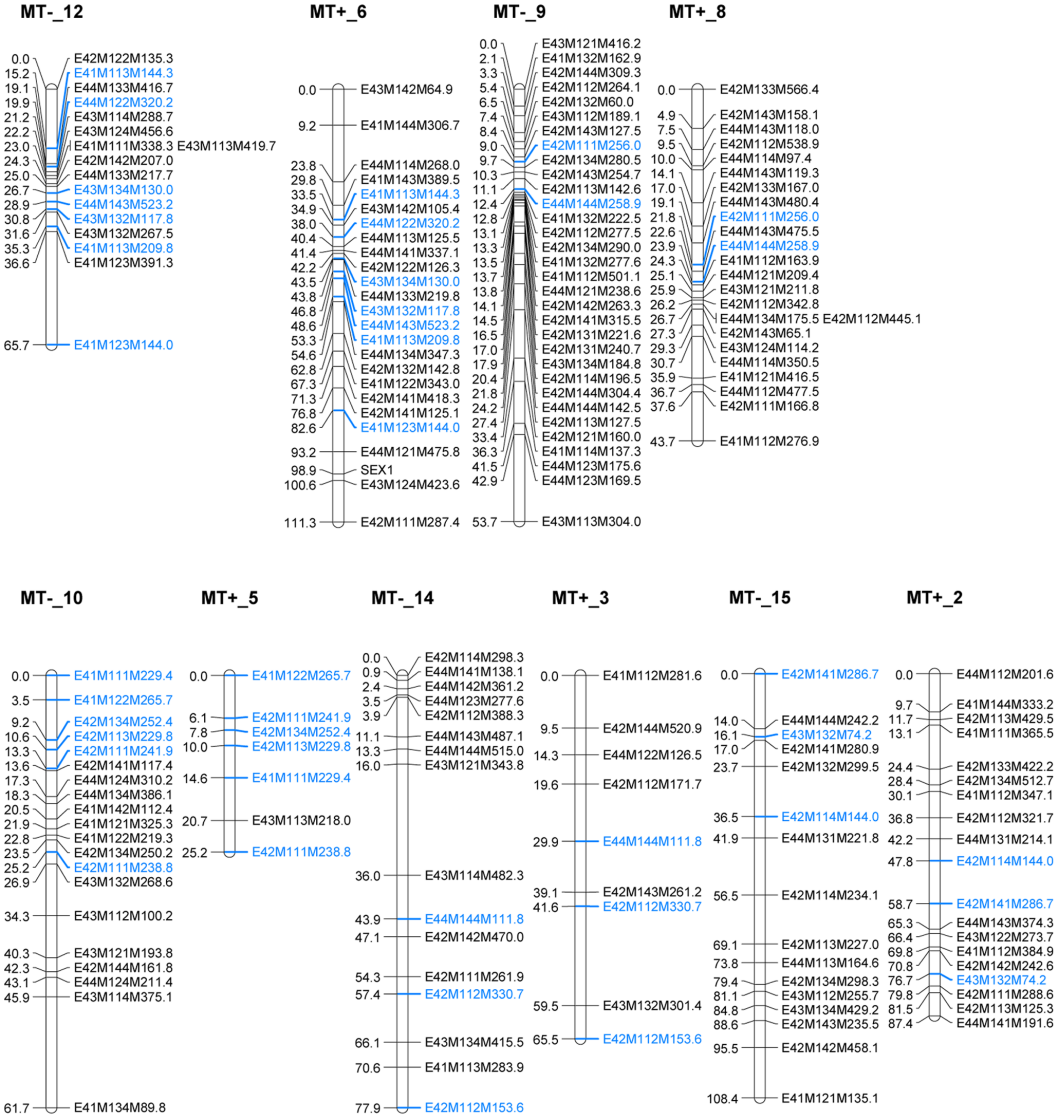
Homologous linkage groups of *S. robusta*. Common biparental markers between the MT^+^ and the MT^−^ linkage groups are shown in blue.

Sixty-four biparental markers were mapped to the MT^+^ and MT^−^ map separately and 20 of those markers showed cosegregation with MT^+^ and MT^−^ specific linkage groups. Five homologous maps were identified based on the presence of one or more identical biparental markers (Figure 3). No significant difference was observed between the intervals of the 20 biparental markers in the MT^+^ and MT^−^ linkage groups (paired *t*-test, *t* = 0.79; *P* = 0.22), suggesting that recombination frequencies do not differ much between both mating types in *S. robusta*.

### Mapping the mating type locus

The mating type phenotype was mapped as a single marker segregating as either a MT^+^ (SEX1) or MT^−^ specific marker (SEX2). SEX1 could be assigned to the MT^+^_6 linkage group, including 18 markers and spanning 115.3 cM. SEX1 is flanked by E43M124_M423.6 at 1.7 cM and E44M121_M475.8 at 5.8 cM distance. In contrast, SEX2 did not cosegregate with any of the markers of the MT^−^ linkage groups (highest LOD  = 2).

**Figure 4:**
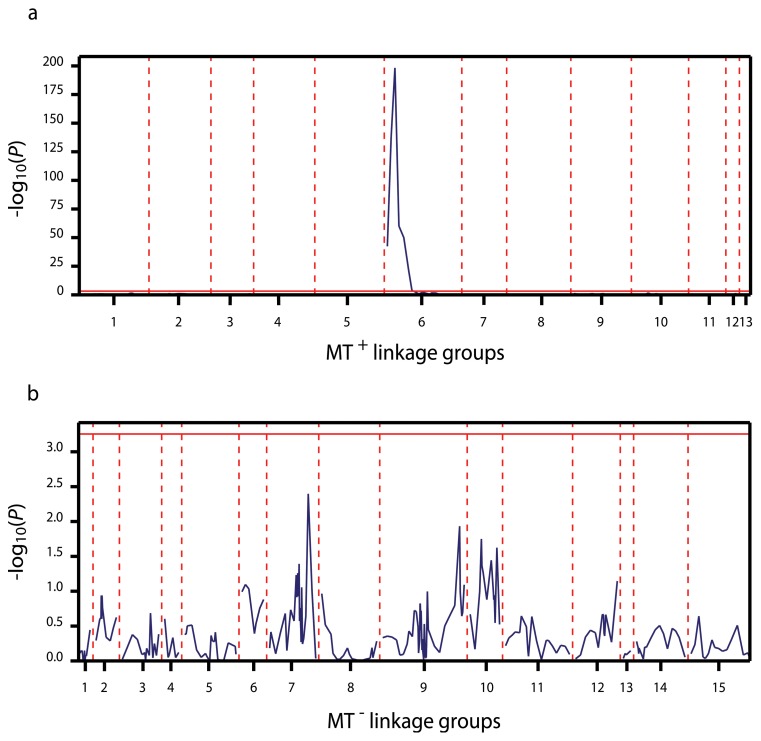
QTL mapping of the mating type phenotype in 116 *S. robusta* F_1_ progeny. QTL analysis was done for the two mating type specific linkage maps separately. Linkage scores (plotted as −log_10_(*P*)) for MT^+^ (a) and MT^−^ (b) markers are shown according to genome position. The linkage analysis indicates that a single locus on the MT^+^_6 linkage group determines the mating type in *S. robusta*.

A QTL analysis of the mating type phenotype confirmed the monogenic nature of mating type and the identification of MT^+^ as the heterogametic sex in *S. robusta* (Figure 4). The genome-wide significance threshold (*P* = 0.05) for detection of QTL co-segregating with mating type was calculated as −log_10_(*P*)  = 3.21. The mating type phenotype mapped significantly (−log_10_(*P*)  = 198.29; E43M124M423.6) to a single locus located on the MT^+^_6 linkage group (Figure 4a). In contrast, no significant association was identified between the mating phenotype and MT^−^ specific markers. (Figure 4b).

The two homologous linkage groups MT^−^_12 and MT^+^_6 (Figure 3) contain six biparental markers widely spread across the two linkage groups. These identify a relatively large region of recombination between the two sex homologous chromosomes.

## Discussion

In this study we exploit the high multiplex ratio of AFLP technology to construct the first linkage maps for a diatom species. We applied these maps to demonstrate that sex determination in the heterothallic pennate species *S. robusta* is genetic, and identify the sex determining region as a single locus.

Segregation and linkage analysis of the 463 AFLP markers scored for 116 individuals of an F_1_ mapping population resulted in 13 MT^+^ and 15 MT^−^ specific linkage groups. The use of biparental markers, segregating in a 1∶2∶1 mode and scored co-dominantly, allowed the detection of five putative homologous linkage groups, including those carrying the mating type locus.

The mating type phenotype cosegregates with markers of a MT^+^ specific linkage group, identifying MT^+^ as the heterogametic sex and MT^−^ as the homogametic sex.

Unlike ‘classical’ sex chromosomes (like the XY chromosomes of mammals and WZ chromosomes of birds), which have only a small recombining pseudo-autosomal region (PAR) or do not recombine at all (e.g. the *Drosophila* Y chromosome), the homologous sex linkage groups in *S. robusta* appear to have a relatively large region of recombination. It is therefore likely that its X and Y chromosomes are cytologically indistinguishable (or non-heteromorphic, cf. Bergero and Charlesworth [29]). Sex chromosomes with extensive recombining PAR regions (in which both homologue chromosomes carry the same gene content) and small non-recombining regions, have been hypothesized to represent recently evolved sex chromosome systems. However, few estimates are available for the age of non-recombining regions (e.g. <2 MY ago in the papaya plant *Carica papaya*), and some data suggest that such regions may also be much older (e.g. in some bird and snake species) [29]. Studies on divergence times in the non-recombining regions of the sex chromosomes in *S. robusta* and other diatoms can contribute to our understanding of the evolution of sex chromosomes, as the diatoms, having an extensive fossil record, have a relatively well time-calibrated evolutionary record. Heterothally is to date known only in the pennate diatoms, which in the fossil record appeared in the Late Cretaceous (about 75 MY ago) [30]. All studied centric diatoms, from whom the pennate diatoms evolved, appear to be homothallic (i.e. they have no mating type differentiation). In some cases, clones are predominantly male or female, but homothally is still present [7]. While more studies on mating systems in centric diatoms are still needed to confirm that homothally is the rule in centric diatoms, all available evidence so far suggests that in diatoms the evolution of heterogametic sex determination associated with mating type differentiation is a relatively recent event, coinciding with the transition from homothally to heterothally.

The first diatom linkage maps presented in this study will constitute an important resource for future genetic analyses in *S. robusta*. Linkage maps provide important insights into genome organization and can be used for genetic studies of traits of interest [31]. A particular advantage is that each individual progeny of the F_1_ mapping population can be clonally propagated and they are maintained as cryopreserved strains. A panel of immortalized F_1_ individuals has a number of advantages for genetic mapping identical to those of Recombinant Inbred Lines (RILs), often used in plant or rodent genetics: one needs to genotype each progeny only once and can phenotype multiple individuals from each clonal culture to reduce individual, environmental and measurement variability for multiple traits.

The linkage maps will also provide important information about the *S. robusta* genome sequence which is under construction (A. Bones & T. Brembu, pers. comm.). The completion of the genome sequence will also be the opportunity to further progress on the linkage mapping, as it will represent a source of single nucleotide polymorphisms (SNPs) and insertion/deletion (INDEL) markers for mapping. This will provide a framework to solve the position and order of scaffolds during assembly [31]. This information can be used to construct pseudo-chromosomes by concatenating adjacent supercontigs, and to carry out broad analyses of genome composition.

Despite the rapidly growing amount of diatom genomic information [32], almost nothing is known about the regulation of the unique diatom life cycle. This is changing fast with the introduction of new model diatoms in which the life cycle and sexual reproduction can be reliably manipulated experimentally, including *S. robusta* [3], [14]. Further characterization of the mating type locus will prove crucial for our understanding of regulation of the diatom life cycle. The identification of the *S. robusta* genomic region carrying the mating type locus in this study provides a starting point for further fine-mapping of the locus and the identification of the gene(s) and sequence polymorphism(s) underlying mating type dimorphism in *S. robusta*. In turn, this will pave the road for understanding the mechanisms underlying mating system switches to alternative reproductive modes (homothally, paedogamy, and apomixis), which are regularly observed among closely related pennate diatoms [12], [15], [33], and more broadly, the evolution of the MT locus in diatoms following the evolution of the pennate lineage from a homothallic centric ancestor.

## Supporting Information

Table S1 **List of primer combinations used for AFLP analysis; E:** ***Eco*** **RI primer with two selective bases; M:** ***Mse*** **I primer with three selective bases, selective bases: 1,2,3,4 corresponds to A, C, G, T.**
(DOCX)

Table S2 **AFLP markers underlying the single QTL detected for the MT phenotype.**
(DOCX)
