# Supplementary material for: Linkage Mapping Identifies the Sex Determining Region as a Single Locus in the Pennate Diatom Seminavis robusta
Source: PLoS One. 2013 Mar 20;8(3):e60132. doi: 10.1371/journal.pone.0060132 (PMC3603935; doi:10.1371/journal.pone.0060132)
Supplement: Table S1 — List of primer combinations used for AFLP analysis; E: Eco RI primer with two selective bases; M: Mse I primer with three selective bases, selective bases: 1,2,3,4 corresponds to A, C, G, T. (DOCX) [file pone.0060132.s001.docx]

| E41M111 | E42M111 | E43M112 | E44M112 |
| --- | --- | --- | --- |
| E41M112 | E42M112 | E43M113 | E44M113 |
| E41M113 | E42M113 | E43M114 | E44M114 |
| E41M114 | E42M114 | E43M121 | E44M121 |
| E41M121 | E42M121 | E43M122 | E44M122 |
| E41M122 | E42M122 | E43M123 | E44M123 |
| E41M123 | E42M131 | E43M124 | E44M124 |
| E41M124 | E42M132 | E43M132 | E44M131 |
| E41M131 | E42M133 | E43M134 | E44M133 |
| E41M132 | E42M134 | E43M142 | E44M134 |
| E41M134 | E42M141 | E43M144 | E44M141 |
| E41M141 | E42M142 |  | E44M142 |
| E41M142 | E42M143 |  | E44M143 |
| E41M143 | E42M144 |  | E44M144 |
| E41M144 |  |  |  |
