# Supplementary material for: Linkage Mapping Identifies the Sex Determining Region as a Single Locus in the Pennate Diatom Seminavis robusta
Source: PLoS One. 2013 Mar 20;8(3):e60132. doi: 10.1371/journal.pone.0060132 (PMC3603935; doi:10.1371/journal.pone.0060132)
Supplement: Table S2 — AFLP markers underlying the single QTL detected for the MT phenotype. (DOCX) [file pone.0060132.s002.docx]

| Marker | Linkage group | Position (cM) | -log10(*P*) |
| --- | --- | --- | --- |
| E42M111M287.4 | MT^+^_6 | 0 | 42.41 |
| E43M124M423.6 | MT^+^_6 | 12.2 | 198.29 |
| E44M121M475.8 | MT^+^_6 | 18.7 | 60.11 |
| E42M141M125.1 | MT^+^_6 | 34.5 | 21.05 |
| E42M141M418.3 | MT^+^_6 | 39.8 | 3.7 |
